# Neither T-helper type 2 nor Foxp3^+^ regulatory T cells are necessary for therapeutic benefit of atorvastatin in treatment of central nervous system autoimmunity

**DOI:** 10.1186/1742-2094-11-29

**Published:** 2014-02-06

**Authors:** Martin S Weber, Thomas Prod’homme, Sawsan Youssef, Shannon E Dunn, Lawrence Steinman, Scott S Zamvil

**Affiliations:** 1 Department of Neurology, University of California, 675 Nelson Rising Lane NS-215A, San Francisco, CA 94158, USA; 2 Program in Immunology, University of California, 675 Nelson Rising Lane, NS-215A, San Francisco 94158, USA; 3 Department of Neuropathology, University Medical Center, Georg August University, Robert-Koch-Str. 40, 37075 Göttingen, Germany; 4 Department of Neurology, University Medical Center, Georg August University, Göttingen, Germany; 5 Department of Neurology and Neurological Sciences, Interdepartmental Program in Immunology, Stanford University, Stanford, CA 94305, USA

## Abstract

Oral atorvastatin has prevented or reversed paralysis in the multiple sclerosis (MS) model experimental autoimmune encephalomyelitis (EAE), and reduced development of new MS lesions in clinical trials. Besides inhibiting development of encephalitogenic T cells, atorvastatin treatment of EAE has been associated with an induction of anti-inflammatory myelin-reactive T-helper type (Th)-2 cells. To investigate the clinical significance of atorvastatin-mediated Th2 differentiation, we first evaluated atorvastatin treatment in interleukin (IL)-4 green fluorescent protein-enhanced transcript (4-GET) reporter mice. Atorvastatin treatment failed to induce IL-4-producing Th2 cells *in vivo*; however, when T cells from atorvastatin-treated 4-GET mice were reactivated *in vitro*, T cells preferentially differentiated into Th2 cells, while antigen-specific T-cell proliferation and secretion of proinflammatory cytokines (interferon gamma, IL-17, tumor necrosis factor and IL-12) were reduced. Oral atorvastatin also prevented or reversed EAE in signal transducer and activator of transcription 6-deficient (STAT6^−/−^) mice, which cannot generate IL-4-producing Th2 cells. Further, atorvastatin treatment did not induce or expand Foxp3^+^ regulatory T cells in either wild-type or STAT6^−/−^ mice. *In vivo* proliferation of T cells, as measured by incorporation of bromodeoxyuridine, was inhibited in atorvastatin-treated wild-type and STAT6^−/−^ mice. These data imply that atorvastatin ameliorates central nervous system autoimmune disease primarily by inhibiting proliferation of proinflammatory encephalitogenic T cells, and not simply through induction of anti-inflammatory Th2 cells. This cytostatic effect may be a relevant mechanism of action when considering use of statins in MS and other inflammatory conditions.

## Introduction

Statins are inhibitors of the enzyme 3-hydroxy-3-methylglutaryl coenzyme A (HMG-CoA) reductase that are widely prescribed to lower serum cholesterol [1]. Besides their metabolic properties, statins attracted interest for their immunomodulatory potential [2]. Statins are clinically beneficial in various models of autoimmune diseases, such as experimental arthritis [3], experimental autoimmune uveoretinitis [4], experimental autoimmune myocarditis [5,6], experimental systemic lupus erythematosus [7] and experimental autoimmune encephalomyelitis (EAE) [2,8-10], the animal model for multiple sclerosis (MS). Based on its potent effect in EAE [11,12], oral statin treatment has been evaluated alone or in combination with established drugs in clinical MS trials [13-16]. In the first placebo-controlled trial testing a statin as monotherapy in MS, atorvastatin (AT) significantly reduced the risk of developing new magnetic resonance imaging demyelinating lesions in patients with clinical isolated syndromes, but did not meet its primary endpoint that included reduction in conversion to clinically definite MS [17,18].

Mechanistically, statins mediate pleiotropic effects on various cells of the immune system [12]. In EAE, clinical benefit mediated by AT treatment is associated with a decreased expression of MHC class II molecules on antigen-presenting cells and a reduced proliferation and T-helper type (Th)-1 differentiation of myelin-reactive T cells [2]. Statin-mediated immune modulation is not due to cholesterol lowering and instead is attributed to the inhibition of post-translational prenylation of small GTP-binding proteins such as Ras, Rac and Rho [19]. Prenylation, a pathway branch for which HMG-CoA reductase is also the rate-limiting enzyme, is required for cell membrane anchoring and proper function of these GTP-binding proteins involved in activation and differentiation of immune cells. Downstream products of these regulatory proteins form activator protein-1, which coordinates with other transcription factors to induce interferon gamma (IFNγ) transcription [20,21]. Statin-mediated inhibition of protein prenylation therefore probably explains suppression of Th1-mediated autoimmunity [19] as reported consistently from animal models. Statins were furthermore shown to target multiple interleukin (IL)-17 regulatory cytokines, leading also to an impaired development of Th17 cells [22]. In many reports investigating statins in various autoimmune settings, treatment was also associated with the occurrence of Th2 cells, whereas some studies reported clinical benefit without development of an anti-inflammatory Th2 phenotype [3,23]. Whether statins solely inhibit encephalitogenic T-cell differentiation or whether they may actively induce regulatory T-cell populations such as Th2 cells or Foxp3^+^ Tregs, considered a desirable goal in treatment of MS, thus remains to be investigated.

In this study we evaluated the kinetics of Th2 differentiation following *in vivo* AT treatment. We also tested the clinical relevance of AT-mediated Th2 differentiation by AT treatment of signal transducer and activator of transcription 6 (STAT6)-deficient mice, which cannot generate IL-4-secreting Th2 cells [24]. AT treatment ameliorated EAE in STAT6-deficient mice, indicating that its clinical effects were not necessarily mediated through induction of Th2 cells. Our further analysis demonstrated that AT prevented expansion of encephalitogenic T cells *in vivo*, suggesting that the cytostatic effects of statins could contribute prominently to their benefit in treatment of central nervous system (CNS) autoimmune disease.

## Materials and methods

### Mice

C57BL/6 female mice, 5 to 8 weeks of age, were purchased from the Jackson Laboratory (Bar Harbor, MN, USA). STAT6-deficient C57BL/6 mice [24] were obtained from SJ Khoury (Harvard University, Cambridge, MA, USA). B10.PL myelin basic protein Ac1-11-specific T-cell receptor transgenic mice [25] were kindly provided by VK Kuchroo (Harvard University) and backcrossed to B10.PL IL-4 green fluorescent protein-enhanced transcript (4-GET) mice [26], which were obtained from R Locksley (University of California, San Francisco, CA, USA). All breeding and experiments were reviewed and approved by the UCSF Institutional Animal Care and Use Committee (Approval number AN077596) and followed the National Institutes of Health guidelines for experimental research on animals.

### Peptides

Rat myelin basic protein peptide Ac1-11 (Ac-ASQKRPSQRHG) was synthesized and purified (>99%) by Quality Control Biochemicals, Inc. (Hopkinton, MA, USA). Mouse myelin oligodendrocyte glycoprotein peptide 35 to 55 (MEVGWYRSPFSRVVHLYRNGK) was synthesized and purified (>99%) by Auspep (Parkville, Australia).

### Experimental autoimmune encephalomyelitis induction

Eight-week-old to 12-week-old female mice were used in all EAE experiments. Mice on the B10.PL background were injected subcutaneously with 100 μg myelin basic protein Ac 1–11 in 0.1 ml phosphate-buffered saline emulsified in an equal volume of complete Freund’s adjuvant supplemented with 2 mg/ml *Mycobacterium tuberculosis* H37RA on day 0 (DIFCO Laboratories, Detroit, MI, USA). In C57BL/6 and C57BL/6 STAT6-deficient mice, EAE was induced by immunization with 25 μg myelin oligodendrocyte glycoprotein peptide 35 to 55 in complete Freund’s adjuvant. After immunization and 48 hours later, mice received an intravenous injection of 300 ng pertussis toxin in 0.2 ml phosphate-buffered saline. Individual animals were evaluated daily, and clinical scores were assessed in a blinded fashion as follows: 0 = no clinical disease, 1 = loss of tail tone only, 2 = mild monoparesis or paraparesis, 3 = severe paraparesis, 4 = paraplegia and/or quadraparesis, and 5 = moribund or death.

### Atorvastatin treatment

AT (prescription formulation; Pfizer, Inc.) was brought into suspension in phosphate-buffered saline as described previously [2]. AT (1 mg/kg/day, 5 mg/kg/day or 10 mg/kg/day) was administered orally in 0.5 ml once daily using a 20 mm feeding needle (Popper and Sons, Inc., New York, USA) starting either 2 days prior to immunization (prevention) or after mice developed a clinical score ≥2 (reversal). Purified AT, used for *in vitro* studies, was provided by Pfizer, Inc.

### Assessment of proliferation

*Ex vivo* proliferative responses were measured using splenocytes 12 days after immunization. Spleen cells (5 × 10^5^) were cultured in 0.2 ml RPMI medium supplemented with 5 × 10^–5^ M 2-mercaptoethanol, 2 mM glutamine, 100 μg/ml penicillin, and 100 μg/ml streptomycin. After 72 hours, cultures were pulsed with 1 μCi [^3^H]-thymidine and harvested 16 hours later. Mean counts per minute of [^3^H] thymidine incorporation were calculated for triplicate cultures. *In vivo* proliferation was evaluated by injection of bromodeoxyuridine (BrdU). Then 200 μl of a 10 mg/ml BrdU solution were injected intraperitoneally 24 hours before evaluation of BrdU incorporation by fluorescent-activated cell sorting (FACS) using a BrdU Flow kit (Pharmingen, San Diego, CA, USA).

### Evaluation of T-cell differentiation

For *in vitro* evaluation of Th1 and Th2 differentiation, naïve (Th0) cells were isolated from B10.PL 4GET T-cell receptor mice by negative selection for CD3 using a MACS separation system (Miltenyi Biotec Boston, MA, USA). T cells were activated by plate-bound anti-CD3 (0.5 μM) and anti-CD28 (1 μM) in the presence of 50 U/ml IL-2 and various concentrations of AT. Addition of 50 μg/ml anti-IFNγ (XMG 1.2) and 50 ng/ml mouse IL-4 or of 20 μg/ml anti-IL-4 and 5 ng/ml IL-12 was used as positive control or negative control for Th2 differentiation, respectively. IL-4-reporting GFP production of T cells was evaluated by FACS. For *ex vivo* analysis, splenocytes or lymph node cells were obtained from B10.PL 4GET T-cell receptor mice 12 days after immunization. Single cell preparations were evaluated for IL-4-reporting GFP production of T cells by FACS staining for CD3. As a control, Th2 differentiation was determined in parallel by intracellular FACS staining for IL-4 (eBioscience, San Diego, CA, USA) after 5 hours of stimulation with PMA phorbol 12-myristate 13-acetate and ionomycin. Induction of CD4^+^CD25^+^FoxP3^+^ Tregs was evaluated using a FACS staining kit by eBioscience.

### Cytokine analysis

Culture supernatants were collected for cytokine analysis at various time points: 48 hours (IL-12), 72 hours (tumor necrosis factor, IFNγ, IL-17, transforming growth factor beta), and 120 hours (IL-4 and IL-10). Enzyme-linked immunosorbent assay (ELISA) was performed using paired monoclonal antibodies specific for corresponding cytokines following the manufacturer’s recommendations (Pharmingen). The results for ELISA assays are expressed as an average of triplicate wells ± standard error of the mean. The SOFTmax ELISA plate reader and software were used for data analysis (Molecular Devices Corporation, Sunnyvale, CA, USA).

### Histopathology

Brains and spinal cords were fixed in 10% formalin. Sections were stained with hematoxylin and eosin. Meningeal and parenchymal inflammatory lesions were counted as described previously [27].

### Statistical analysis

Data are presented as the mean ± standard error of the mean. For clinical scores, significance between groups was examined using the Mann–Whitney *U* test; *P* < 0.05 was considered significant. All other statistical analysis was performed using a one-way multiple-range analysis of variance test for multiple comparisons; *P* < 0.01 was considered significant.

## Results

### Atorvastatin treatment promotes development of Th2 cells *in vitro*, but not *in vivo*

**Figure 1:**
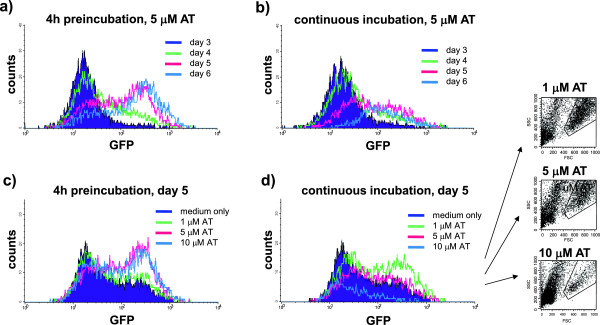
**T-helper type 2 cell differentiation upon *in vitro *atorvastatin treatment.** Naïve T cells isolated from transgenic interleukin (IL)-4-reporter (green fluorescent protein (GFP)-enhanced transcript) mice were incubated with 1, 5 or 10 μM atorvastatin (AT) either 4 hours prior to stimulation (preincubation; **(a), (c)**) or continuously while stimulated (**(b), (d)**) with 0.5 μg/ml αCD3 and 1 μg/ml αCD28. Expression of IL-4-reporting GFP was evaluated by fluorescence-activated cell sorting at the time-point indicated. (d) Expansion of T cells was visualized by forward scatter (FSC)–side scatter (SSC). Shown is one representative out of three independent experiments.

To evaluate the kinetics of AT-mediated induction of Th2 cells, we tested AT treatment in mice expressing IL-4 linked via a viral IRES element with enhanced green fluorescent protein (eGFP). These IL-4 4-GET reporter mice were shown to faithfully report the evolution of IL-4-expressing Th2 cells *in vivo*[26,28]. Using Th2-polarizing cytokines, we first confirmed that naïve T cells isolated from 4-GET mice differentiate into eGFP^+^ T cells *in vitro* (Additional file 1: Figure S1). We then tested the ability of AT to promote Th2 differentiation of naïve T cells upon αCD3/αCD28 stimulation. As shown in Figure 1a, 4-hour preincubation with 5 μM AT facilitated the development of IL4-producing Th2 cells when these cells were subsequently stimulated in media not containing AT. In contrast, continuous exposure to the same dose during αCD3/αCD28 stimulation inhibited expansion of naïve T cells, indicating that Th2 differentiation primarily occurred upon recovery from statin exposure (Figure 1b). As shown in Figure 1c, preincubation of naïve T cells with 1 μM, 5 μM and 10 μM AT led to a dose-dependent Th2 differentiation as evaluated 5 days after stimulation. Upon continuous exposure to the same doses (Figure 1d), 1 μM AT promoted a Th2 bias whereas higher doses again impaired T-cell expansion.

**Figure 2:**
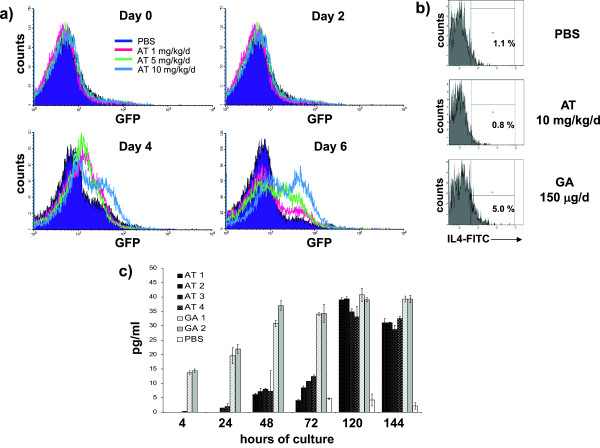
**T-helper type 2 cell differentiation occurs *in vitro *following *in vivo *atorvastatin treatment. (a)** Interleukin (IL)-4-reporter (green fluorescent protein (GFP)-enhanced transcript) mice were fed daily with 1, 5 or 10 mg/kg/day atorvastatin (AT) for 12 days starting 2 days prior to immunization with myelin basic protein Ac1-11. Splenic T cells were washed, taken into culture and evaluated daily for production of IL-4-reporting GFP. *Ex vivo* IL-4 production by T cells was also evaluated by **(b)** intracellular cytokine staining (day 0, gated on CD4^+^ T cells) and **(c)** enzyme-linked immunosorbent assay (supernatants taken at the time points indicated); mice injected subcutaneously daily with 150 μg glatiramer acetate (GA) in phosphate-buffered saline (PBS) served as positive control. Five mice/group were used. Shown is one representative finding out of three independent experiments performed. FITC, fluorescein isothiocyanate.

To evaluate the effect of oral AT treatment *in vivo*, 4-GET mice were treated daily with 1 mg/kg/day, 5 mg/kg/day or 10 mg/kg/day AT starting 2 days prior to immunization. These doses have been previously shown to prevent EAE [2]. As shown in Figure 2a (day 0), eGFP^+^ Th2 cells were not detectable in splenocytes isolated from AT-treated 4-GET mice. It remained possible that a minor population of IL-4-producing Th2 cells were generated in response to *in vivo* AT treatment, but were not detected by this method. We therefore also measured the intracellular level of IL-4 by T cells by flow cytometry. AT treatment at 10 mg/kg/day did not promote *in vivo* transcription of IL-4 in T cells detectable by this method either (Figure 2b). As a positive control, mice were treated daily with glatiramer acetate (GA) at a dose of 150 μg, which is known to promote development of Th2 cells *in vivo*[29]. Similarly, IL-4 secretion was evident by ELISA in freshly isolated T cells from GA-treated mice, but not T cells from AT-treated mice (Figure 2c). As lymphocytes had been re-stimulated with antigen ex *vivo* in previous studies that observed Th2 polarization with AT treatment, we also examined T cells from AT-treated 4-GET mice after various times of antigen re-stimulation *ex vivo* in the absence of AT. To evaluate whether Th2 deviation of T cells may occur as a secondary effect following *in vivo* AT treatment, splenocytes obtained from *in vivo* AT-treated mice were taken into culture. As shown in Figure 2a, without *in vitro* stimulation, T cells isolated from AT-treated mice but not from control-treated mice developed into eGFP^+^, IL-4-producing Th2 cells starting on day 4. The extent of this *in vitro* Th2 deviation correlated with the dose of AT used for *in vivo* treatment and occurred with a maximum after 6 days of *in vitro* culture. These results were confirmed when IL-4 secretion by *in vitro* cultured T cells isolated from AT-treated and GA-treated mice was compared by ELISA (Figure 2c).

### Atorvastatin treatment prevents and reverses EAE in STAT6-deficient mice

**Figure 3:**
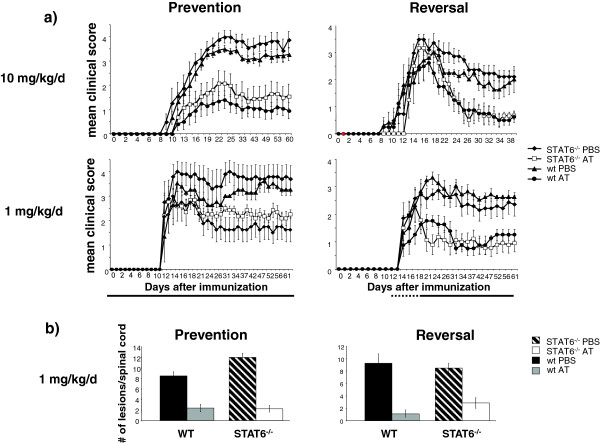
**STAT6-deficient C57Bl/6 mice are fully protected by atorvastatin treatment.** Signal transducer and activator of transcription 6 (STAT6)-deficient and wild-type (WT) C57Bl/6 mice were fed daily with atorvastatin (AT; 1 mg/kg/day or 10 mg/kg/day) or vehicle (phosphate-buffered saline (PBS)) starting either 2 days prior to immunization (prevention, left panels) or after experimental autoimmune encephalomyelitis (EAE) was fully established (reversal, right panels). **(a)** Mice were evaluated daily for clinical EAE symptoms. **(b)** At study termination, mice were sacrificed and evaluated for histologic signs of EAE. Shown is the number of inflammatory lesions per three representative (cervical, thoracal, lumbal) hematoxylin and eosin-stained spinal cord sections. Twelve mice/group were used; two independent studies were performed for each setting.

Because we did not observe Th2 polarization during *in vivo* AT treatment, we evaluated whether STAT6-dependent development of Th2 cells was required for the clinical benefit mediated by AT treatment of EAE. Here, we investigated whether AT treatment was effective in STAT6-deficient mice, which are incapable of generating IL4-producing Th2 cells [24]. Although STAT6-deficient mice developed slightly more severe EAE than control-treated wild-type mice [24] (Figure 3a), *in vivo* AT treatment both prevented EAE and reversed paralysis in STAT6-deficient mice, comparable with its effect in wild-type mice (Figure 3a). Similar results were obtained in wild-type or STAT6-deficient mice treated with oral AT at a dose of 1 mg/kg/day, the equivalent of the highest US Food and Drug Administration-approved dose of 80 mg/day in humans (Figure 3a). As shown in Figure 3b, oral AT (1 mg/kg/day) reduced the number of CNS inflammatory lesions in a similar manner in of STAT6-deficient mice and wild-type mice. Reduction in CNS inflammation occurred when treatment started prior to immunization (left panel) or after disease was established (right panel).

**Figure 4:**
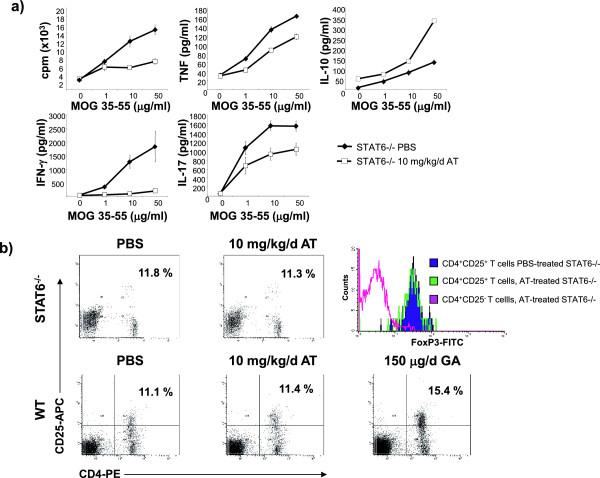
**Atorvastatin treatment inhibits proliferation, Th1 and Th17 differentiation of myelin-reactive T cells but does not expand CD4**^**+**^**CD25**^**+**^**FoxP3**^**+ **^**regulatory T cells.** Splenocytes were isolated from signal transducer and activator of transcription 6 (STAT6)-deficient or wild-type (WT) C57BL/6 mice dosed daily with atorvastatin (AT; 10 mg/kg/day) or vehicle (phosphate-buffered saline (PBS)) for 12 days starting 2 days prior to immunization with myelin oligodendrocyte glycoprotein peptide 35 to 55 (MOG 35–55). **(a)** Cytokine secretion in response to MOG 35–55 was evaluated by enzyme-linked immunosorbent assay. **(b)** The frequency of CD4^+^CD25^+^FoxP3^+^ regulatory T cells (Tregs) was evaluated by fluorescence-activated cell sorting. As positive control, mice were injected subcutaneously with 150 μg glatiramer acetate (GA) in PBS per day. Shown is the percentage of CD4^+^CD25^+^ Tregs within all CD4^+^ T cells. Five mice/group were used. Shown is one representative finding out of two independent experiments performed. cpm, counts per minute; FITC, fluorescein isothiocyanate; IFN, interferon; IL, interleukin; PE, phycoerythrin; TNF, tumor necrosis factor.

AT treatment of wild-type mice has been associated with inhibition of T-cell proliferation and reduction in proinflammatory cytokine production [2,8]. As shown in Figure 4a, AT treatment of STAT6-deficient mice was also associated with reduction of both T-cell proliferation and secretion of tumor necrosis factor, IFNγ and IL-17, while, as expected, IL-4 was not detectable. These results confirm that the beneficial effect of AT treatment does not require STAT6 signaling. Interestingly, AT treatment was associated with increased IL-10 secretion. However, it was not associated with an increase in CD4^+^CD25^+^FoxP3^+^ Tregs in either STAT6-deficient or wild-type mice. As a positive control, and consistent with previous observations [29-32], GA-treatment promoted Treg expansion in mice (Figure 4b).

### Atorvastatin treatment inhibits T-cell proliferation *in vivo*

**Figure 5:**
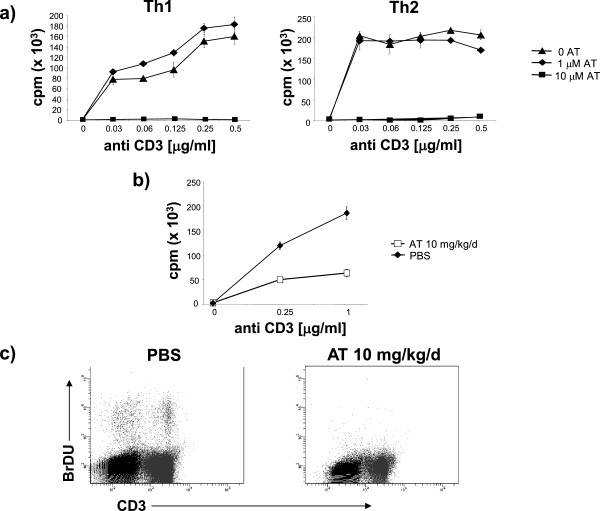
**Atorvastatin treatment inhibits proliferation of T cells *in vivo*. (a)** Atorvastatin (AT) dose-dependently inhibits proliferation of both differentiated T-helper type (Th)-1 and Th2 cells. Naïve T cells from interleukin (IL)-4-reporter (IL-4 green fluorescent protein-enhanced transcript (4GET)) mice were stimulated with 0.5 μg/ml αCD3 in the presence of 20 μg/ml αIL-4 and 5 ng/ml IL-12 (Th1 condition, left panel) or 50 μg/ml α-interferon (IFN)-γ and 50 ng/ml IL-4 (Th2 condition, right panel). Respective polarization of T cells was confirmed at day 5. Differentiated T cells were then washed and re-stimulated with the indicated concentrations of αCD3 in the presence of 0, 1 or 10 μM AT. T-cell proliferation was assessed by H_3_ incorporation. Shown is one representative out of three independent experiments. **(b)** T cells isolated from IL-4-reporter (4GET) mice treated with 10 mg/kg/day AT for 12 days starting 2 days prior to immunization were evaluated for proliferation following activation with the indicated dose of αCD3. **(c)** IL4-reporter (4GET) mice were immunized with myelin basic protein Ac 1–11. Starting 2 days prior to immunization, mice were fed daily with 10 mg/kg/day AT for 12 days. Twenty-four hours before T-cell isolation, mice were injected intraperitoneally with 200 μl of a 10 mg/ml bromodeoxyuridine (BrdU) solution. BrdU incorporation of freshly isolated, splenic T cells was evaluated by fluorescence-activated cell sorting staining for CD3 and BrdU. Five mice/group were used. Shown is one representative finding out of three independent experiments performed. cpm, counts per minute.

We observed that *in vivo* AT treatment during EAE inhibited proliferation of myelin-specific T cells upon re-stimulation *ex vivo*[2,8] (Figure 4a), suggesting that these myelin-reactive cells expanded less in AT-treated mice post vaccination. A reduction in expansion of myelin-reactive T cells would also explain the lowered proinflammatory cytokine production observed by these cells. Thus, we further explored the anti-proliferative effects of AT *in vitro* and *in vivo. In vitro*, AT inhibited the proliferation of both differentiated Th1 and Th2 cells (Figure 5a); and following *in vivo* AT treatment, T cells exhibited a reduced ability to proliferate after *ex vivo* stimulation with anti-CD3 and anti-CD28 (Figure 5b). To evaluate whether oral AT treatment inhibited T-cell proliferation *in vivo* in these disease settings, we administered mice with BrdU, a pyrimidine nucleotide analogue that is incorporated during DNA synthesis and reflects cell division. As shown in Figure 5c, CD3^+^ T cells from AT-treated mice showed almost no BrdU incorporation at 12 days post vaccination, thus confirming that AT treatment had a potent anti-proliferative effect on T cells *in vivo*.

## Discussion

AT and other cholesterol-lowering statins have been shown to be clinically beneficial in various models of T-cell-mediated autoimmune disease. These studies have consistently reported that statin treatment inhibits proinflammatory Th1 cytokine production. Despite the use of various statins and differences in dose or route of administration, many – but not all [3,6,23] – of these studies attributed the benefit of statin treatment in autoimmune models to development of Th2 cells [2,5,33,34]. Here we used IL4-reporter mice and STAT6-deficient mice as tools to further explore the involvement anti-inflammatory Th2 cells in the anti-inflammatory effects of AT. We found that dosing of AT *in vivo* failed to promote detectable frequencies of Th2 cells, but T cells isolated from AT-treated mice did produce IL-4 in increased amounts after re-stimulation in culture in the absence of AT. Similar results were obtained when myelin-specific T cells were treated with AT *in vitro*; pre-incubation of naïve T cells prior to activation led to a dose-dependent development of Th2 cells, whereas continuous exposure at higher doses inhibited T-cell activation and differentiation. The anti-proliferative effect may thus be a dominant effect of AT. Furthermore, we found that despite their inability to generate IL-4 producing T cells, STAT6-deficient mice were fully protected from development of proinflammatory Th1 and Th17 cells and clinical EAE by AT treatment. Our results clearly demonstrate that Th2 polarization is not required for the *in vivo* benefit of statin treatment in CNS autoimmune disease. Finally, we provide strong evidence that the anti-proliferative effect of AT probably accounts for a good proportion of its effects in dampening CNS autoimmunity.

By inhibiting HMG-CoA reductase, statins interfere with the rate-limiting step in the mevalonate pathway that generates cholesterol and isoprenoid derivatives, including farnesyl-pyrophosphate and all-trans geranylgeranyl-pyrophosphate. When studying T-cell differentiation *in vitro* using farnesyl-pyrophosphate and all-trans geranylgeranyl-pyrophosphate, as well as selective antagonists, we previously demonstrated that farnesylation of the small GTP binding protein, Ras, is required for Th1 differentiation and that geranylgeranylation of RhoA is necessary for T-cell proliferation [19]. As statins prevent production of both farnesyl-pyrophosphate and all-trans geranylgeranyl-pyrophosphate, they can inhibit both T-cell differentiation and proliferation, respectively. As we did not observe Th1, Th2 or Th17 differentiation *in vivo* during AT treatment, we questioned whether AT also exerted a potent anti-proliferative effect *in vivo*. Here, we observed that 1 mg/kg oral AT treatment, a dose approximately equivalent (weight/weight) to the 80 mg dose administered in recent clinical MS trials, nearly shut down T-cell proliferation *in vivo* in mice. These results, along with the therapeutic effect in STAT6-deficient mice and our inability to demonstrate Th2 polarization at the time of AT treatment, suggest that the cytostatic effect of statin treatment may provide the predominant mechanism responsible for observed benefits in MS clinical trials.

In our investigation, we observed that AT treatment inhibited T-cell proliferation upon re-stimulation *in vitro*, and did so in an antigen nonselective manner. Our data are consistent with the work of Aktas and colleagues [8], who demonstrated that AT mediated inhibition of T-cell proliferation *in vitro*, which was linked to downregulation of cyclin-dependent kinase 4. Interestingly, a predominant anti-proliferative effect rather than induction of Th2 cells could also explain the benefit of statin treatment in Th2-mediated and B-cell-mediated models of allergic asthma [35] and systemic lupus erythematosus [7], respectively.

While our data demonstrate that Th2 polarization is not required for the beneficial immunomodulatory effect of statin treatment, they do not exclude the contribution of statin-induced anti-inflammatory cytokines. In this regard, the clinical benefit mediated by AT treatment was associated with an increased secretion of IL-10. There are a number of immune cell populations that express IL-10, including B regulatory cells and anti-inflammatory macrophages. Future studies will address which immune cell(s) other than T cells may also be producers of IL-10 in AT-treated mice. Interestingly, our results are reminiscent of findings from one of the first monotherapy MS trials. In that MS trial, high-dose AT treatment led to a reduction in the number and volume of newly emerging CNS lesions, and was associated with an enhanced secretion of IL-10, but not IL-4 [36].

Data indicate that there is a reduction in Treg number and function in MS. Restoration of the Treg balance appears to be an important mechanism of action of GA in MS [29,30,37,38]. Interestingly, there are only limited data suggesting that statins may promote expansion of Tregs [39-41]. We found that neither treatment initiated prior to disease induction nor following EAE onset altered the frequency of CD4^+^CD25^+^Foxp3^+^ Tregs in diseased mice. A potential increase in Tregs is therefore unlikely to significantly contribute to the anti-inflammatory effect of statins in CNS autoimmune disease.

How does this new information impact the potential use of statins in MS [42]? At first glance, nonspecific inhibition of proinflammatory T-cell differentiation in MS may be viewed less advantageously than the active induction of an anti-inflammatory T-cell phenotype [17,18,43]. Interestingly, a recent clinical trial in secondary progressive MS further suggests that simvastatin may slow progression and that this effect also occurs largely independent of peripheral immune modulation [44]. Taken together, these trials demonstrate that statin treatment is beneficial at various stages in the disease. One strategy may be to use statins in combination with approved MS drugs. Several recent trials have investigated the combination of statins with IFNβ [13-16]. While results are not unequivocal [45], two of these studies suggested that addition of oral statin to IFNβ therapy may result in a paradoxical increase of MS activity [13-16]. In general, for two agents to exert synergistic benefit, distinct mechanisms of action are desirable, while employing the same mechanism may increase the risk of antagonism [46]. Statins and GA have complementary mechanisms of action [47]. Whereas GA promotes induction of both Th2 cells and Tregs [29,30,37], statins have been associated with a Th2 bias in treatment of CNS autoimmunity and, as we have shown in this study, a prominent anti-proliferative effect. In EAE, this combination had a synergistic beneficial effect [47]. However, clinical trials will be necessary to determine whether such a combination will elicit a clinical beneficial effect in MS.

One may exercise caution when testing statins in combination with more recently approved MS therapeutic agents. In this regard, it is important to recognize that by inhibiting *de novo* pyrmidine synthesis, teriflunomide has a primary cytostatic effect on proliferating lymphocytes [48,49]. While the clinical benefit of dimethyl fumarate has been attributed to its activation of the antioxidant transcription factor (erythroid-derived 2)-related factor (*Nrf2*) pathway [49,50], it also exerts anti-proliferative activity [51]. Given the strong *in vivo* anti-proliferative effects of AT, one might envisage testing a short course of high-dose oral statin as an adjunct to, or *in lieu* of, high-dose intravenous or oral steroids for MS exacerbations.

## Abbreviations

AT: Atorvastatin; BrdU: Bromodeoxyuridine; CNS: Central nervous system; EAE: Experimental autoimmune encephalomyelitis; eGFP: Enhanced green fluorescent protein; ELISA: Enzyme-linked immunosorbent assay; FACS: Fluorescent-activated cell sorting; GA: Glatiramer acetate; 4-GET: Green fluorescent protein-enhanced transcript; HMG-CoA: 3-hydroxy-3-methylglutaryl coenzyme A; IFN: Interferon; IL: Interleukin; MS: Multiple sclerosis; STAT6: Signal transducer and activator of transcription 6; Th: T-helper type; Treg: Regulatory T cell.

## Competing interests

The authors declare that they have no competing interests.

## Authors’ contributions

All authors read and approved the final manuscript. MSW performed all experiments and wrote the manuscript. TP, SD and SY analysed data. LS and SSZ designed the experiments and wrote the manuscript.

## Supplementary Material

Additional file 1: Figure S1 Showing naïve T cells isolated from transgenic IL-4-reporter (4-GET) mice stimulated with 0.5 μg/ml αCD3 and 1 μg/ml αCD28 in the presence of 50 μg/ml anti-IFNγ and 50 ng/ml mouse IL-4 (Th2 condition), 20 μg/ml anti-IL-4 and 5 ng/ml IL-12 (Th1 condition) or without any supplementation (neutral). Expression of IL-4-reporting green fluorescent protein (GFP) was evaluated by FACS 5 days after stimulation. Shown is one representative out of five independent experiments. Click here for file
