# Supplementary figures and images for: Neither T-helper type 2 nor Foxp3+ regulatory T cells are necessary for therapeutic benefit of atorvastatin in treatment of central nervous system autoimmunity
Source: J Neuroinflammation. 2014 Feb 6;11:29. doi: 10.1186/1742-2094-11-29 (PMC3922392; doi:10.1186/1742-2094-11-29)

## Slide 1
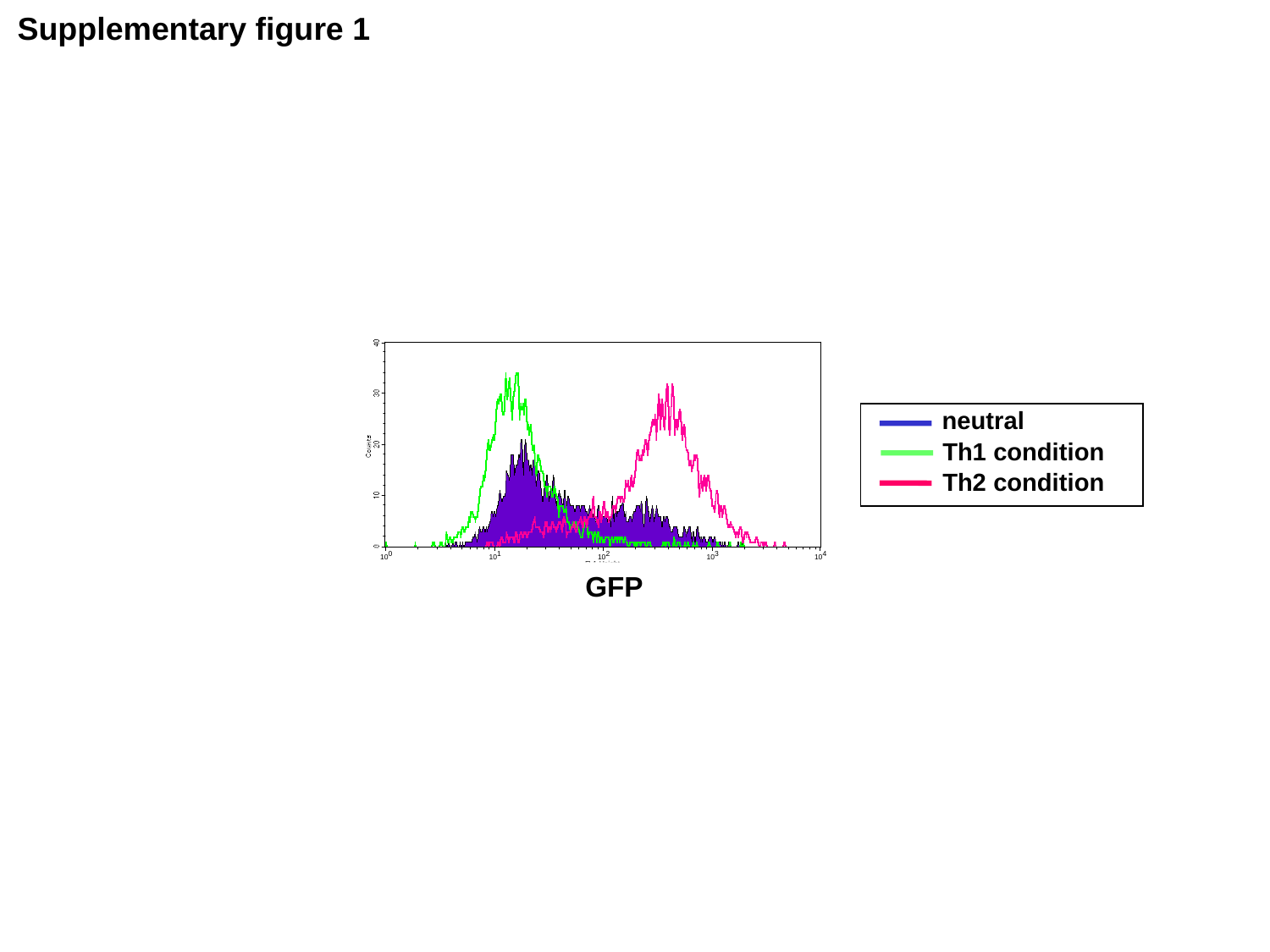

Supplementary figure 1
neutral
Th1 condition
Th2 condition
GFP

Supplement: Additional file 1: Figure S1 — Showing naïve T cells isolated from transgenic IL-4-reporter (4-GET) mice stimulated with 0.5 μg/ml αCD3 and 1 μg/ml αCD28 in the presence of 50 μg/ml anti-IFNγ and 50 ng/ml mouse IL-4 (Th2 condition), 20 μg/ml anti-IL-4 and 5 ng/ml IL-12 (Th1 condition) or without any supplementation (neutral). Expression of IL-4-reporting green fluorescent protein (GFP) was evaluated by FACS 5 days after stimulation. Shown is one representative out of five independent experiments. [file 1742-2094-11-29-S1.ppt]
